# A curated crowdsourced dataset of Luganda and Swahili speech for text-to-speech synthesis

**DOI:** 10.1016/j.dib.2025.111915

**Published:** 2025-07-23

**Authors:** Andrew Katumba, Sulaiman Kagumire, Joyce Nakatumba-Nabende, John Quinn, Sudi Murindanyi

**Affiliations:** a Department of Electrical and Computer Engineering, Makerere University, Kampala, Uganda; b Department of Computer Science, Makerere University, Kampala, Uganda

**Keywords:** Speech dataset, Text-to-speech, Low-resource languages, Luganda, Kiswahili

## Abstract

This data article describes a curated, crowdsourced speech dataset in Luganda and Kiswahili, created to support text-to-speech (TTS) development in low-resource settings. The dataset is derived from Mozilla’s Common Voice corpus and includes only validated utterances from female speakers. A multi-step curation process was used to enhance the consistency and quality of the data. Speakers were first manually selected based on similarities in intonation, pitch, and rhythm, then validated using acoustic clustering with pitch features and mel-frequency cepstral coefficients (MFCCs). Audio files were preprocessed to remove leading and trailing silences using WebRTC voice activity detection, denoised with a causal waveform-based DEMUCS model, and filtered using WV-MOS, an automatic speech quality scoring tool. Only clips with a predicted MOS score of 3.5 or higher were retained. The final dataset contains over 19 h of Luganda and 15 h of Kiswahili recordings from six female speakers per language, each paired with a text transcription. This dataset is designed to support speech generation research in Luganda and Kiswahili and enable reproducible experimentation in end-to-end TTS systems.

Specifications Table

**Table:** 

Subject	Computer Sciences
Specific subject area	Text-to-speech data for low-resource African languages (Luganda and Kiswahili)
Type of data	Audio, CSV
Data Collection	Speech recordings and their transcriptions were sourced from Mozilla Common Voice (Luganda v12.0 and Swahili v15.0) [1]. Only validated clips from female speakers were selected. Audio was preprocessed using WebRTC Voice Activity Detection (for trimming silences), a causal DEMUCS model (for denoising), and WV-MOS scoring (to filter low-quality clips). Transcriptions were included as text entries in accompanying CSV files, along with metadata such as speaker ID and clip duration.
Data source location	Institution: Makerere University City: Kampala Country: Uganda
Data accessibility	Repository name: Mendeley Data Data identification number: 10.17632/nb8b25h9nj.3 Direct URL to data: https://data.mendeley.com/datasets/nb8b25h9nj/3
Related research article	Author’s name: Katumba, A., Kagumire, S., Nakatumba-Nabende, J., Quinn, J. and Murindanyi, S. Title: Building Text-to-Speech Models for Low-Resourced Languages From Crowdsourced Data Journal: Applied AI Letters, DOI: https://doi.org/10.1002/ail2.117

## 1 Value of the Data

• This data provides a curated and ready-to-use set of speech recordings and transcriptions for Luganda and Kiswahili, enabling the development of TTS or speech systems in settings with limited existing resources.
• Researchers and developers working on speech synthesis, multilingual NLP, or voice technologies for African languages can benefit from using this dataset.
• The dataset supports experiments on multi-speaker TTS, speaker consistency, and the effectiveness of crowdsourced speech for neural synthesis models.
• The data is already cleaned, denoised, and quality-filtered, making it suitable for direct use in training end-to-end TTS models.

## 2 Background

The motivation behind compiling this dataset was to support the development of text-to-speech (TTS) models for low-resource African languages, particularly Luganda and Kiswahili. Existing TTS systems typically rely on large single-speaker studio-quality corpora, which are expensive and often unavailable for many underrepresented languages. This dataset was constructed to explore whether carefully selected and preprocessed crowdsourced data could serve as a practical alternative. The audio and transcripts were sourced from Mozilla’s Common Voice platform, a public initiative that collects multilingual speech from volunteers. A pipeline was developed to select consistent speakers, apply noise reduction, trim silence, and filter for audio quality. The dataset includes audio from six female speakers per language and is intended to be directly usable in end-to-end neural TTS pipelines.

This dataset was used in the accompanying article [2], which investigates synthesis using crowdsourced data. This data article provides the full curated dataset used in that study, along with detailed documentation and a reproducible format for use by other researchers working on speech synthesis in low-resource settings.

## 3 Data Description

This dataset comprises curated and preprocessed speech recordings and corresponding transcriptions in Luganda and Kiswahili, aimed at supporting research in low-resource text-to-speech (TTS) synthesis. The data was extracted from the validated splits of Mozilla Common Voice Luganda (v12.0) and Kiswahili (v15.0), then passed through a multi-step pipeline to ensure consistency, clarity, and suitability for TTS modeling. The dataset includes 7842 Luganda utterances totaling approximately 19.2 h and 6324 Kiswahili utterances totaling approximately 15.1 h of audio.

The dataset is organized into two main folders, Luganda/ and Kiswahili/, each containing:

• wavs.zip: a ZIP archive of .wav audio clips from six female speakers, denoised, trimmed, and filtered for quality.
• metadata.csv: a file listing each audio file’s name and corresponding transcription.

All audio clips are in 16-bit mono .wav format at a sampling rate of 22.05 kHz. Each utterance is stored in an individual audio file. Metadata files include two columns: **filename** and **transcript**.

## 4 Experimental Design, Materials, and Methods

This dataset was derived from Mozilla Common Voice (CV) Luganda (v12.0) and Kiswahili (v15.0), which are among the few open-source resources available for African languages. Unlike well-established TTS datasets such as LJSpeech (a 13-hour single-speaker English corpus recorded in studio conditions) and CSS10 (a multi-language, single-speaker dataset for 10 languages), Common Voice provides diverse, crowdsourced, multi-speaker recordings that are not originally optimized for TTS. Despite their variability, Common Voice datasets offer a promising foundation for scalable TTS development in low-resource settings, provided that careful preprocessing and quality filtering are applied.

Prior studies have explored how Common Voice can be repurposed for TTS. Van Niekerk et al. [6] trained competitive English TTS models by filtering Common Voice data for quality and consistency. Giraldo et al. [7] similarly developed Catalan TTS datasets using perceptual quality metrics like NISQA and speech enhancement tools such as VoiceFixer. Our work builds on these approaches and adapts them to African language constraints, introducing a multi-stage pipeline focused on speaker consistency, audio quality, and reproducibility. Key improvements include a hybrid speaker selection method combining manual listening and acoustic clustering, waveform-level denoising using DEMUCS [4], and speech quality filtering using WV-MOS, rather than traditional SNR or ASR-based heuristics. These steps are tailored to address the unique challenges of working with crowdsourced African language data and to ensure that the resulting dataset is suitable for TTS model training.

### 4.1 Speaker selection

We identified the top 20 female speakers for each language based on utterance count and manually reviewed 5 samples per speaker. Human evaluators ranked speakers by consistency in pitch, rhythm, and clarity. Six speakers whose voices were unanimously preferred were selected. Acoustic features including pitch, energy, MFCCs, and rhythm were extracted and clustered using k-means to confirm that selected speakers were acoustically distinct and internally consistent.

### 4.2 Audio preprocessing

**Fig. 1:**
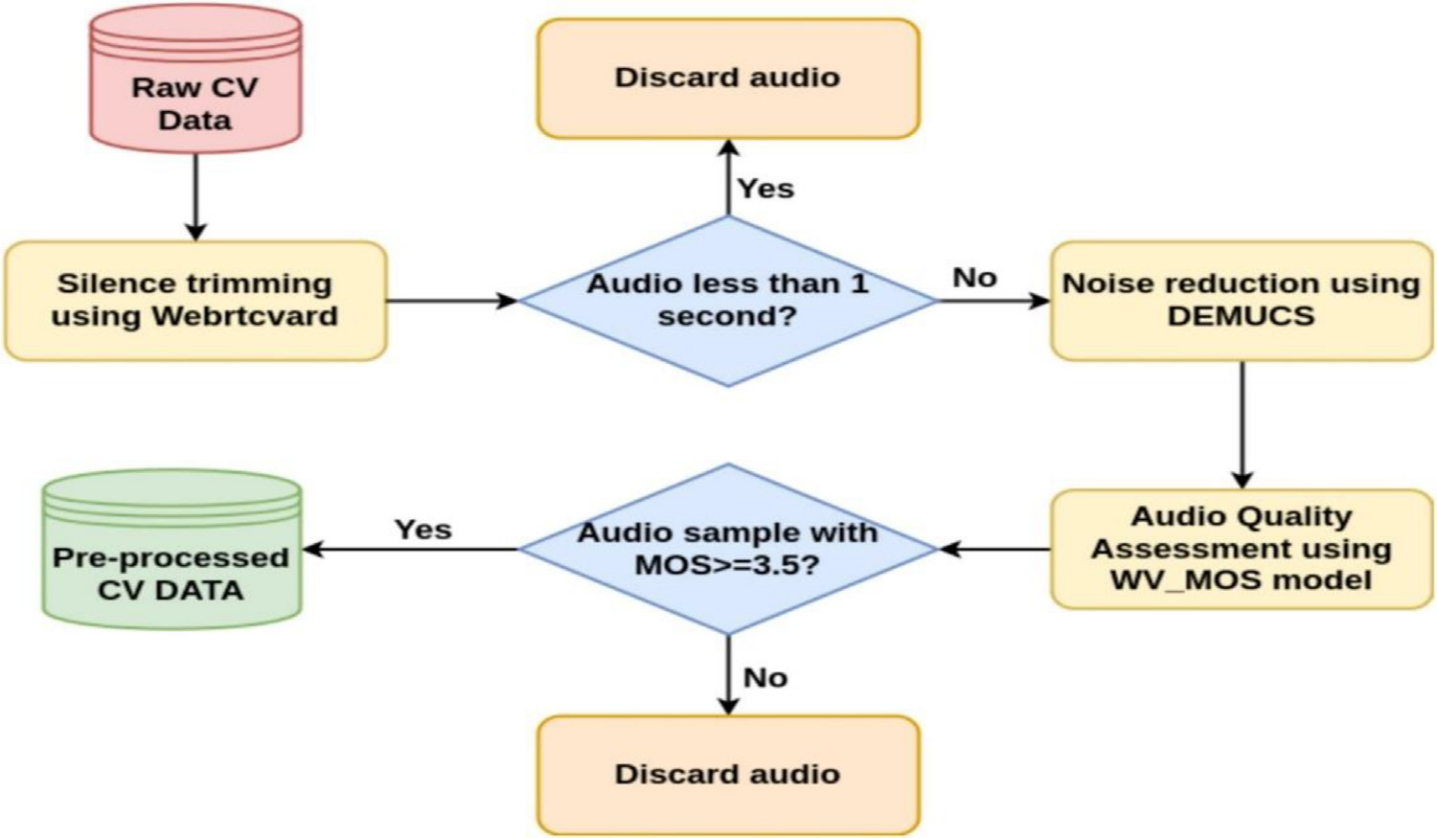
Overview of the Luganda and Kiswahili TTS dataset curation pipeline.

We applied a multi-stage preprocessing pipeline as shown in Fig. 1.

1. Silence trimming: We used WebRTC Voice Activity Detection (VAD) to remove leading and trailing silence. This method is commonly used in telephony and speech applications to isolate active speech regions while preserving key acoustic cues. We applied a 90 % voiced-frame threshold and 300 ms padding to reduce clipping artifacts.
2. Noise removal: A real-time version of the DEMUCS model was applied for waveform-level denoising. This causal encoder-decoder model is optimized for removing both stationary and non-stationary background noise while maintaining speech integrity. DEMUCS has demonstrated strong performance in enhancing raw waveform quality for ASR and speech enhancement tasks, especially under noisy conditions [6].
3. Quality filtering: We used WV-MOS, a wav2vec-based model that predicts Mean Opinion Scores (MOS), to automatically assess the perceptual quality of each utterance. Only samples scoring ≥3.5 were retained. WV-MOS correlates well with human judgments and has been used in TTS and voice cloning pipelines to ensure high fidelity.

Fig. 1 illustrates an overview of the dataset curation pipeline. The diagram illustrates the main stages in the preprocessing workflow after speaker selection: silence trimming using WebRTC VAD, noise reduction using DEMUCS, and quality filtering with WV-MOS. The result is a curated, high-quality dataset of multi-speaker speech suitable for training text-to-speech models in low-resource languages.

### 4.3 Data structure

Each utterance is stored as a mono .wav file sampled at 16 kHz. The filenames follow the Common Voice naming convention. Each audio clip is paired with a row in a CSV file containing filename and transcript fields. The structure is consistent across both languages. A sample of the dataset content is shown in Table 1.

**Table 1:** Sample excerpts from each language’s metadata.

	Filename	Description
Luganda	common_voice_lg_23,717,981.wav	Nze nnakoowa okukima amazzi kuba temunsasula buli lwe ngakima.
	common_voice_lg_23,718,760.wav	Neebaza nnyo ssezaala wange kuba yangabira ente.
	common_voice_lg_23,718,761.wav	Ntudde nnyo kati omugongo gunnuma.
Kiswahili	common_voice_sw_28,905,667.wav	Pia mara nyingi aliandika katika magazeti.
	common_voice_sw_28,902,728.wav	Pia katika upishi na kutengeneza vyakula mbalimbali kama aisikrimu.
	common_voice_sw_28,905,313.wav	Makao makuu ya eneohilo ni Kouassi-Kouassikro.

### 4.4 Tools and code

We used open-source tools for all steps in the pipeline:

• WebRTC VAD [3]
• DEMUCS for noise removal [4]
• WV-MOS model [5]

No manual annotations or proprietary software were used. The entire preprocessing pipeline can be reproduced using standard Python libraries and the referenced open-source tools.

## Limitations

This dataset is derived from Mozilla Common Voice, which is a crowdsourced platform originally designed for automatic speech recognition, not for text-to-speech. As such, several limitations exist. First, the recording conditions are uncontrolled, which introduces variation in background noise, microphone quality, and speaking environments. Although denoising and quality filtering were applied, some variability may remain. Second, the dataset comprises only short utterances (1–30 s), limiting its suitability for training models that require longer, prosodically rich input. Lastly, the Kiswahili and Luganda portions of the Common Voice dataset differ in overall recording quality, which may reflect in downstream modeling outcomes

## Ethics Statement

The authors confirm that they have read and comply with the ethical requirements for publication in *Data in Brief*. This dataset does not involve human subjects, animal experiments, or data collected from social media platforms. All speech recordings were obtained from Mozilla Common Voice, a public, open-source platform in which contributors voluntarily donate their voice data under a Creative Commons license. The data used in this work were already publicly available and anonymized, and no personally identifiable information is included.

## Credit Author Statement

**Andrew Katumba:** Conceptualization, Supervision, Funding acquisition, Writing - Review & Editing. **Sulaiman Kagumire:** Data curation, Software, Formal analysis, Methodology, Investigation, Writing – Original Draft, Visualization, Writing - Review & Editing. **Joyce Nakatumba-Nabende:** Conceptualization, Supervision, Funding acquisition, Writing - Review & Editing. **John Quinn:** Conceptualization, Supervision, Funding acquisition, Investigation. **Sudi Murindanyi:** Investigation, Writing – Original Draft, Visualization, Writing - Review & Editing.

## Data Availability

Mendeley DataA Curated Crowdsourced Dataset of Luganda and Swahili Speech for Text-to-Speech Synthesis (Original data).
